# Gut Microbiota and Infectious Complications in Advanced Chronic Liver Disease: Focus on Spontaneous Bacterial Peritonitis

**DOI:** 10.3390/life13040991

**Published:** 2023-04-11

**Authors:** Valeria Maccauro, Carlo Airola, Francesco Santopaolo, Antonio Gasbarrini, Francesca Romana Ponziani, Maurizio Pompili

**Affiliations:** 1 Internal Medicine and Gastroenterology-Hepatology Unit, Fondazione Policlinico Universitario Agostino Gemelli IRCCS, 00168 Roma, Italy; 2 Department of Translational Medicine and Surgery, Catholic University, Largo Francesco Vito 1, 00168 Roma, Italy

**Keywords:** cirrhosis, spontaneous bacterial peritonitis, gut-liver axis, microbiota, intestinal barrier, bacterial translocation

## Abstract

Liver cirrhosis is a chronic disease that can be complicated by episodes of decompensation such as variceal bleeding, hepatic encephalopathy, ascites, and jaundice, with subsequent increased mortality. Infections are also among the most common complications in cirrhotic patients, mostly due to a defect in immunosurveillance. Among them, one of the most frequent is spontaneous bacterial peritonitis (SBP), defined as the primary infection of ascitic fluid without other abdominal foci. SBP is mainly induced by Gram-negative bacteria living in the intestinal tract, and translocating through the intestinal barrier, which in cirrhotic patients is defective and more permeable. Moreover, in cirrhotic patients, the intestinal microbiota shows an altered composition, poor in beneficial elements and enriched in potentially pathogenic ones. This condition further promotes the development of leaky gut and increases the risk of SBP. The first-line treatment of SBP is antibiotic therapy; however, the antibiotics used have a broad spectrum of action and may adversely affect the composition of the gut microbiota, worsening dysbiosis. For this reason, the future goal is to use new therapeutic agents that act primarily on the gut microbiota, selectively modulating it, or on the intestinal barrier, reducing its permeability. In this review, we aim to describe the reciprocal relationship between gut microbiota and SBP, focusing on pathogenetic aspects but also on new future therapies.

## 1. Introduction

Liver cirrhosis is the 10th leading cause of death in industrialized countries [1]. In this setting, despite the reduction in viral hepatitis spread, alcohol consumption and nonalcoholic fatty liver diseases (NAFLD) are increasing causes of advanced chronic liver disease (ACLD), leading to a dramatic peak in mortality rate during the last three decades [2]. Evidence suggests that liver damage from inflammation to fibrosis is progressive and partially reversible, until the breakpoint of portal hypertension is reached [3]. After that, in decompensated patients with clinically significant portal hypertension, the mortality rate is about 57% [4]. Decompensation is characterized by serious life-threatening complications, such as variceal bleeding, hepatic encephalopathy, and ascites [5]. Furthermore, people with cirrhosis have a high risk for development of infections and sepsis, which is almost doubled when compared with patients hospitalized for other diseases [5]. Such an increased susceptibility is related to an acquired immunodeficiency, known as “cirrhosis associated immune dysfunction” (CAID). Indeed, even though immune cells are hyperactivated in the prodromal phases of cirrhosis, their antibacterial properties are impaired, leading to a condition of cellular and humoral immune dysregulation. Since the intestinal barrier is of paramount importance in preventing infections in cirrhotic patients, gut microbiota has gained an increase in attention. Indeed, the loss of intestinal barrier integrity is thought to be associated with CAID, as the persistent translocation of a huge number of bacteria and their products fuels this chronic and dysfunctional inflammatory condition [6,7]. Moreover, the gut is the gateway through which bacteria can easily translocate into bloodstream and other sterile fluids, aided by the depression of immune defenses. These mechanisms are implicated in initiating and maintaining the primary infection of the ascitic fluid, named spontaneous bacterial peritonitis (SBP), which represents the leading cause of infection in patients with cirrhosis and is responsible of a four-fold increase in mortality in decompensated patients [8]. Studies on the gut microbiota, focusing on its contribution to chronic inflammation and progression of ACLD, suggest its implication also in the development of SBP [9]. In this review, we aim to describe the role of the gut–liver axis in modulating the risk of infections in patients with liver cirrhosis, analyzing then the relationship between the gut microbiota and SBP, with a special focus on novel therapies.

## 2. Infections and Liver Cirrhosis

Infections are a major cause of cirrhosis decompensation and globally increase mortality fourfold [10,11]. Approximately two-thirds of patients with cirrhosis and extrahepatic organ failure present with sepsis; in-hospital mortality in cirrhotic patients with severe sepsis exceeds 75%, and the risk of sepsis-related death due to multi-drug resistant bacteria is currently increasing as new therapies for such microorganisms are still not available, representing an unsolved public health concern [12,13]. The higher infectious risk of cirrhotic patients could be related to specific molecular and cellular mechanisms associated with liver dysfunction, but also with other host-related factors (e.g., old age, malnutrition, immunosuppressive drugs use) and with the gut microbiota. So far, the balanced interaction between the host and the gut microbiota has been considered a protective factor against all infection types in cirrhotic patients. In fact, gut microbiota is critical in providing resistance against colonization by exogenous microorganisms, and dysbiosis has been associated with a higher risk of infection [14]. Gut microbiota analysis performed on cirrhotic patients with nosocomial infections showed a reduction in commensal bacteria and an increase in pathogens [15]. Therefore, immune system, intestinal microenvironment, and host factors are the main actors in modulating the risk of infections in cirrhotic patients.

### 2.1. Immune-Paralysis: Molecular and Cellular Mechanisms

Decompensated cirrhosis is usually characterized by a multi-systemic inflammatory condition, in which immune innate cells are constantly stimulated by pro-inflammatory molecules, such as pathogens-associated molecular patterns (PAMPs) derived from bacteria translocated through the intestinal mucosa. PAMPs trigger the local inflammatory response inducing cellular death, with the consequent release of damage-associated molecular patterns (DAMPs), which, interacting with immune cells, enhance inflammation. On the other side, the immune system sets in place a condition of tolerance, which is known as endotoxin tolerance [16]. Emerging data reported that during bacterial infection associated with decompensated cirrhosis, certain types of monocytes, known as monocytic myeloid-derived suppressor cells (M-MDSCs) and MER-receptor tyrosine kinase (MERTK) cells, migrate to the infection site impairing the potential activation of T cells; indeed, the phenotype of effector CD8+ T cells changes into the exhausted one, which is characterized by increased expression of programmed cell death protein 1 (PD-1) and T cell immunoglobulin and mucin domain-containing protein 3 (TIM-3) [17,18]. In addition, it has been recently demonstrated that liver cirrhosis decompensation is associated with increased blood levels of both soluble stimulating and inhibitory immune checkpoint receptors. Both the immune-stimulating sCD40 receptor and immune-suppressive soluble B and T-cell lymphocyte-associated (sBTLA) receptor seem to be involved, highlighting the ambivalent role of lymphocytes that could change from an effector into an exhausted phenotype [19]. Furthermore, cirrhosis impairs the hepatic reticuloendothelial system, by altering Kupffer cells’ function, inducing sinusoidal capillarization, and resulting in the recruitment of dysfunctional immune cells [20]. In patients with cirrhosis, hepatic synthesis of complement proteins and acute-phase proteins decreases, leading to the impairment of opsonization and the reduction of chemotactic activity, as well as of macrophages phagocytic activity [20]. Large CD14+ CD206+ peritoneal macrophages are another specific subpopulation of immunosuppressive monocytes, which is typical of decompensated cirrhosis. This macrophage population is responsible for the dysregulation of the innate immunity in the ascitic fluid. Recent evidence has demonstrated that during SBP, large peritoneal macrophages secreted a cleaved protein named soluble mannose receptor (sCD206), which is associated with an increased local and systemic inflammatory response [21]. CD206 is typically expressed by M2 macrophages, an anti-inflammatory phenotype of polarized macrophages [22,23]. Several phenotypes of macrophages have been described; among them, M1 and M2 are the most well-known. M1 macrophages principally produce pro-inflammatory cytokines and seem to be mostly involved in acute liver injury [24]. On the other hand, M2 polarization is more related to liver fibrosis/cirrhosis progression [25] and development of complications, such as hepatocellular carcinoma (HCC) [26]. Although the mechanisms driving the M2 polarization are complex and not completely understood, the gut microbiota seems to play a significant role. Dysbiosis can directly induce an M2 phenotype through the interaction between PAMPs and toll-like receptor 4 (TLR4) expressed on macrophage surface [27]. Nonetheless, a balanced macrophage polarization is essential for the organism response to bacterial infections [28]. On the contrary, the loss of this complex equilibrium can lead to a higher rate of infectious disease and is associated with a worse prognosis. Increased blood levels of sCD206 have been proposed as a marker of abnormal M2 polarization and have been related to the severity of some infections, such as community-acquired pneumonia [29]. As a consequence, inflammation is constantly activated but immune cells lose their physiological effectiveness, becoming unable to face pathogenic stimulations and to maintain a homeostatic interaction between the intestinal bacteria and the host. This inability of the immune system seems to be crucially involved in the increased susceptibility to infections of patients with cirrhosis. Due to this immune dysfunction, an apparent unresponsiveness against infections and cellular damage occurs, in a condition known as immune-paralysis [30]. Furthermore, the persistent overflow of PAMPs and their translocation in the systemic circulation contributes to worsening the pre-existing hyperdynamic circulation associated with cirrhosis, leading to uncontrolled systemic vasodilation, impaired cardiac output, and hypotension, with a compensatory hyperactivation of the renin–angiotensin–aldosterone system that also precipitates cirrhosis decompensation [31]. In an attempt to limit infection and inflammation spreading, when sepsis occurs, patients with acute on chronic liver failure (ACLF), IL6, IL17, and IL10 are over-produced, further worsening liver cellular damage and coagulation dysfunction [32].

### 2.2. Infections and Liver Cirrhosis: Clinical Concerns

**Figure 1:**
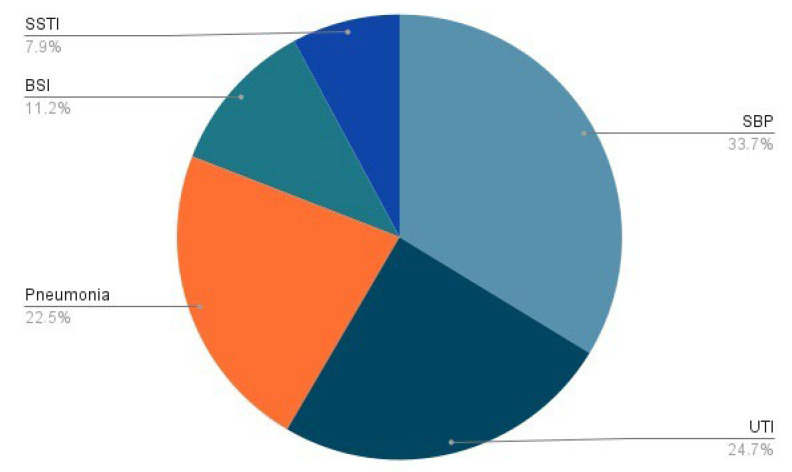
Pie chart showing the prevalence of bacterial infections in hospitalized cirrhotic patients. BSI: spontaneous bloodstream infection; SBP: spontaneous bacterial peritonitis; SSTI: soft skin tissue infection; UTI: urinary tract infection.

About 25–46% of cirrhotic patients are hospitalized for acute decompensation due to bacterial infections, two-third of whom diagnosed at admission (within 48 h): 30–50% are community-acquired, 25–40% are health care-associated, whereas one-third are nosocomial. Even though SBP has been reported to be the most common type of infection, with a prevalence of 25–35%, sites of extra-peritoneal colonization could also lead to life-threatening complications. Among them, 20–25% are represented by urinary tract infections (UTIs), 20% by pneumonia, 8–15% by spontaneous bloodstream infections, and 5–10% by skin and soft tissues infections (SSTIs) [33] (Figure 1). Nosocomial infections often develop after invasive procedures, as central venous and urinary catheters placement or mechanical ventilation, as the disruption of innate immune barriers realizes an entrance gate into the bloodstream for pathogens and commensal bacteria [30]. About half of all infections recognize a bacterial cause, with a prevalence of Gram-negative over Gram-positive strains, while 10–15% of patients suffer from secondary fungal infections [33]. High mortality has also been observed among patients with cirrhosis who have severe acute respiratory syndrome coronavirus 2 (SARS-CoV-2) infection [34]. *Enterobacteriaceae* (such as *Escherichia coli* and *Klebsiella pneumoniae*) and other gram-negative bacteria are the main pathogens in SBP and UTIs (50–70%), with a minor contribution of Gram-positive bacteria as *Staphylococcus aureus* and *Enterococci* (30–45%). On the contrary, pneumonia and SSTIs are more frequently caused by Gram-positive strains. Due to the high prevalence of health care-associated infections, involved bacteria are frequently multi-drug resistant (MDR), as extended spectrum beta lactamase and AmpC-producing *Enterobacteriaceae*, methicillin-resistant *Staphylococcus aureus*, and vancomycin-resistant *Enterococci*, or extensively drug-resistant (XDR), as carbapenemase-producing *Enterobacteriaceae*, carbapenem-resistant *Pseudomonas aeruginosa*, and *Acinetobacter baumannii* [33]. In the last two decades, the proportion of resistant bacteria has increased due to a widespread use of quinolones for SBP prophylaxis and of third-generation cephalosporines for treatment of other nosocomial infections; this represents an important challenge for clinicians, as the risk of antibiotic failure, progression to septic shock, and overall hospital mortality is increased [35].

### 2.3. Clinical Features of SBP

SBP is the main cause of infection in individuals with liver cirrhosis, occurring in 25–35% of decompensated cirrhotic inpatients and in 3.5% of decompensated cirrhotic outpatients, with a survival rate per year of 30–50%. SBP diagnosis is based on the finding of an ascitic polymorphonuclear cell count ≥ 250/microL, with either positive or negative microbiological culture. Aerobic Gram-negative bacteria, such as *Escherichia coli* and *Klebsiella pneumoniae*, are those most frequently implicated [36]. Immunological dysfunction is strongly involved in SBP pathogenesis, and comorbidities can contribute to the immunosuppression promoting SBP onset. Malnutrition is frequent in patients with cirrhosis and has been associated with higher prevalence of SPB [37]. Indeed, lower levels of serum zinc are an independent predictor of hepatic decompensation and SBP [38]. Vitamin D deficiency seems also to be related with an increased risk of SBP in patients with HCV-related cirrhosis [39]. Furthermore, lower serum levels of 25-hydroxyvitamin D could predict a worse outcome in these patients [39]. Other systemic comorbidities usually associated with an immune dysregulation are implicated in SBP development, such as type 2 diabetes, which increases the risk of SBP six-fold in patients with cirrhosis [40]. However, SBP is characterized by the absence of a primary source of intra-abdominal infection. For this reason, alongside the immune system dysregulation, the loss of intestinal mucosal barrier and the consequent gut bacteria translocation are considered to be the main pathogenic mechanism, thus confirming a crucial role of the gut–liver axis.

## 3. The Gut–Liver Axis in the Pathogenesis of SBP

### 3.1. Bacterial Translocation in Liver Cirrhosis

**Figure 2:**
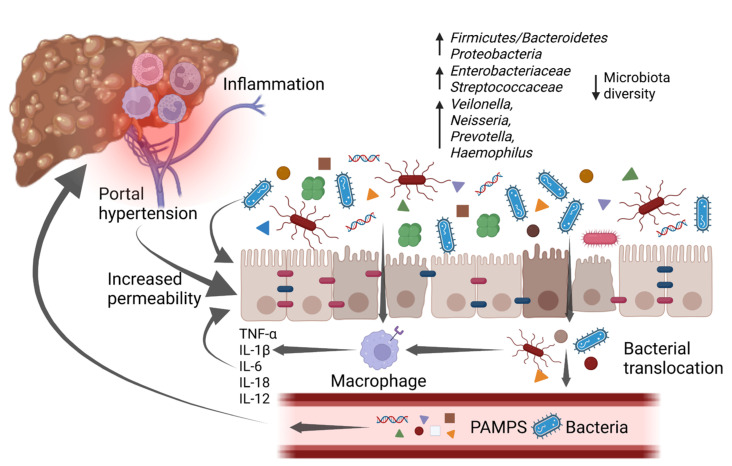
An increase in pro-inflammatory bacteria and a reduction in overall diversity are typical of gut microbiota changes associated with cirrhosis. These changes promote a low-grade chronic inflammatory state that impairs intestinal barrier function, which is already affected by portal hypertension, leading to a condition known as “leaky gut”. The increased intestinal permeability, along with the disrupted immune system, allows bacterial translocation and the increase of bacterial metabolites in the bloodstream. As a consequence, it enhances inflammation which worsens intestinal barrier disruption and in the liver promotes cirrhosis progression, with the emergence of its complications. IL-1β: interleukin-1β; IL-6: interleukin-6; IL-12: interleukin-12; IL-18: interleukin-18; PAMPs: pathogen-associated molecular patterns; TNF-α: tumor necrosis factor α. Created with BioRender.com accessed on 10 January 2023.

Portal hypertension causes a profound dysfunction of the gut–liver axis. The vasodilation of splanchnic vessels reduces blood flow velocity and capacitance in the intestinal mucosa, leading to a chronic ischemic condition. This acts as an inflammatory trigger, inducing fibromuscular proliferation of the intestinal layers and modifying the morphological characteristics of enterocytes [41]. In a descriptive pilot study, intestinal biopsies from a population of cirrhotic patients showed a reduced representation of tight junctions (TJ) proteins such as occludin and claudin 1. Histologically, loss of microvilli and reduced villi to crypt ratio have been evidenced too [41,42]. As a result of the epithelial barrier dysfunction, intestinal permeability is increased, realizing a condition also known as leaky gut, which promotes bacterial translocation and a consequent inflammatory cascade (Figure 2) [7,41]. The release of small amounts of PAMPs, such as endotoxins, lipopolysaccharides (LPS), and bacterial DNA, from the gut into the portal venous system to extra-intestinal sites is a physiological process, which has been described even in healthy subjects, with no clinical consequences. However, in patients with liver cirrhosis, bacterial translocation is exacerbated by several pathological mechanisms such as reduced secretion of defensins and regenerating islet-derived proteins (RegIIIβ and RegIIIγ), thickening of the inner mucus layer by over-expression of MUC2, reduced intestinal intraluminal concentration of secondary bile acids, decreased mucosal IgA secretion, impaired gastric acid secretion, and slower oro-cecal transit time (OCTT) due to ineffective gastrointestinal peristalsis [7,43]. Bacterial translocation underlies immunotolerance in cirrhotic patients; indeed, chronic inflammation induces a dysfunction of the innate and adaptive immune response, which together with the reduced number of circulating leukocytes due to hypersplenism and the reduced production of humoral and complement factor leads to a sort of “immune paralysis” [44]. Moreover, phagocytosis stimulated by the binding of LPS with TLR4 on the macrophage surface is ineffective, and secondly the Kupffer cells activated by bacterial products release multiple cytokines in a sort of “cytokine storm”, enhancing liver damage [45]. The vicious circle that links gut dysbiosis, bacterial translocation, and liver cirrhosis is described in Figure 2.

### 3.2. The Gut Microbiota in Cirrhotic Patients with SBP

Cirrhotic patients typically present qualitative and quantitative alterations of the gut microbiota composition, which worsen with the progression of the disease and hamper intestinal permeability and bacterial translocation [31,46]. Much evidence suggests that cirrhosis-related dysbiosis is characterized by the overabundance of pathogens such as *Enterobacteriaceae* and the depletion of beneficial commensals; furthermore, it is associated with the development of decompensation episodes as well as HCC, and may even identify patients at unfavorable prognosis [47,48,49,50]. Bacterial DNA can be detected in the ascites of half of cirrhotic patients even in the absence of SBP, probably due to bacterial opsonization [44]. A recent study using the amplification of 16S r-DNA demonstrated that, in case of negative culture, ascites from cirrhotic patients contained less bacterial DNA than in case of positive culture (Table 1) [51]. *Enterobacteriaceae*, *Streptococcus pneumoniae*, and *Streptococcus viridans* are the bacterial species most frequently detected in the ascitic fluid of patients with SBP; among them, *Escherichia coli* is reported to be the most abundant, but also Gram-positive bacteria such as *Staphylococcus*, *Streptococcus*, and *Enterococcus*, as well as *Helicobacter* spp., including *Helicobacter pylori*, have been described [51]. Indeed, a remarkable result of the gastrointestinal dysfunction associated with liver cirrhosis is the oralization of the small intestinal and the colonic microbiota. About 48–73% of cirrhotic patients present a small intestinal bacterial overgrowth (SIBO), especially those with previous episodes of SBP or hepatic encephalopathy [52]. A pivotal Chinese study observed that at least half of the stool bacteria species detectable in cirrhotic patients belong to the oropharyngeal inhabitants [46]. This was subsequently confirmed comparing stool samples of 182 cirrhotic patients with healthy controls; in particular, oral species such as *Streptococcus oralis* and *Streptococcus parasanguinis* not only co-existed with enteric species, such as *Enterococcus* spp., *Clostridium* spp., and *Erysipelatoclostridium ramnosum*, but were correlated with disease severity [48]. Given these premises, it is not surprising that proton pump inhibitors are reported to further enrich oral bacteria in the intestine of cirrhotic patients and are associated with readmissions [7,53] and the development of SBP in patients with ascites [54].

A recent study by Bajaj et al. [55] analyzed the metagenomic composition of bacteria and phages from the stools of cirrhotic patients with SBP versus uninfected cirrhotic patients with ascites; a lower alpha and beta diversity was observed in the gut microbiota of patients with SBP, with a higher abundance of *Clostridioides difficile*, *Enterococcus*, *Veillonella* spp., and other potential pathobionts. In contrast, the gut microbiota of uninfected patients was enriched by *Eubacterium*, *Bifidobacterium*, *Ruminococcus*, and *Alistipes* spp., similarly to healthy subjects. Phages intestinal community resembled the characteristics of bacterial one, with a low alpha diversity being observed in uninfected patients; *Bifidobacterium* and *Streptococcus* phages were dominant in un-infected patients, whereas those related to *Escherichia coli*, *Streptococcus*, and *Yersinia* were higher in infected subjects. Overall, infected patients hosted more phages linked to potentially pathogenic species belonging to *Enterococcus faecalis*, *Enterococcus faecium*, and *Clostridioides difficile* along with *Lactobacillus* spp. and oral-origin taxa such as *Veillonella parvula* and *S. parasanguinis* and lower *Bacteroides*, *Alistipes* spp., *Faecalibacterium*, and *Lachnospiraceae* spp., which conversely were enriched in uninfected patients. In addition, phage species analysis revealed no differences in alpha diversity, but only in beta diversity, between infected and uninfected cirrhotic patients, with a predominance of *Escherichia*, *Streptococcus*, and *Campylobacter* phages in the former group. Therefore, authors examined the linkage network between bacteria and phages, showing that in uninfected patients, *E. faecium* was negatively linked to several commensal bacteria, whereas in the infected group, positive correlations between *E. faecium* and Gram-positive taxa, as well as negative linkages between *E. faecium* and commensal species such as *Ruminococcus* spp. and *Bacteroides* spp., were found. In infected patients, phages/bacteria linkages were associated with functional and pathogenic features, conferring biofilm formation ability and antibiotic resistance, and facilitating infection spreading. In patients with SBP, the gut microbiota was composed predominantly by species belonging to *Enterococcus* and *Escherichia* genera, and by their associated phages, with a reduction of commensals *Bifidobacterium*, *Eubacterium*, *Blautia*, *Ruminococcus* spp. and phages linked to them.

Another study by Zhou et al. [56] comparing the stool gut microbiota composition between patients with SBP and healthy controls confirmed that the overall microbial richness was significantly reduced (*p* < 0.001); however, gut microbial diversity was higher in patients with SBP than healthy controls. Comparing patients with SBP to patients without SBP, a higher abundance of *Pantoea*, *Serratia marcescens*, *Klebsiella pneumoniae*, *Prevotella oris*, and *Escherichia coli*, as well as a lower abundance of the *Ruminococcus torques*, *Faecalibacterium prausnitzii*, *Methanobrevibacter smithii*, and *Lactobacillus reuteri* group were observed; these alterations correlated with a worse clinical outcome, as demonstrated by increased white blood cell count, C-reactive protein and procalcitonin serum levels, and a worse Child–Pugh Score. The main studies analyzing the gut microbiota composition in patients with cirrhosis and SBP are resumed in Table 1.

In conclusion, SBP is associated with gut dysbiosis, with an overgrowth of Gram-negative and oral bacteria; thus, gut microbiota modulation with antibiotics or beneficial bacterial strains could be considered as a potential treatment for cirrhotic patients with SBP.

**Table 1:** Studies analyzing gut microbiota composition in cirrhotic patients with spontaneous bacterial peritonitis (SBP). “↑” means an increase; “↓” means a decrease.

Study	Design	Sequencing Method	Sample	Results
Feng et al., 2015 [44]	Retrospective	16S rRNA Pyrosequencing	Ascitic fluid	↑ *Enterobacteriaceae* (*Escherichia coli*), *Streptococcus* *pneumoniae* and *Streptococcus viridans*, *Staphylococcus*, *Enterococcus*, *Helicobacter* spp. (*Helicobacter pylori*)
Mücke et al., 2020 [57]	Prospective	16S rRNA Pyrosequencing	Saliva and Stools	No significant ↓ in Shannon diversity and bacterial richness, ↑ *Enterobacteriaceae* at baseline, ↓ *Enterobacteriaceae* after 12 weeks
Zhou et al., 2022 [56]	Prospective	16S rRNA Pyrosequencing	Stools	↑ Richness ↓ Diversity ↑ *Gammaproteobacteria*, *Proteobacteria*, *Enterobacterales*, *Klebsiella*, *Serratia*, *Acinetobacter*, and *Moraxellaceae*
Bajaj et al., 2022 [55]	Prospective	Metagenomic analysis of bacterial DNA	Stools	↑ *Clostridioides difficile*, *Enterococcus faecalis* and *faecium*, *Veillonella* spp., *Streptococcus* *parasanguinis* ↑ *E. coli*, *Streptococcus*, *Yersinia*, *Campylobacter* phages ↓ *Eubacterium*, *Blautia*, *Faecalibacterium*, *Lachnospiraceae* spp., *Bifidobacterium*, *Ruminococcus*, *Alistipes* spp. ↓ *Bifidobacterium* and *Streptococcus* phages ↑ Gram-positive bacteria (*Lactobacillus*, *Enterococcus*, *Acidaminococcus*), *Akkermansia*, *Pseudomonas*, CrAss phages and *Escherichia*, *Enterococcus*, *Shigella*, *Streptococcus*, *Mycobacterium* phages ↓ Gram negative bacteria

## 4. Effect of SBP Treatment on the Gut Microbiota

The treatment of SBP is represented by antibiotics, which are also recommended as primary prophylaxis in high-risk patients or as secondary prophylaxis, together with intravenous albumin [58,59,60]. A recent study focused on the effect of norfloxacin given in secondary prophylaxis on the gut microbiota, finding a reduction in bacterial alpha diversity and richness that was not statistically significant, as well as negligible changes in the gut microbiota after initiation of antibiotic prophylaxis compared to baseline (*p* > 0.05). *Firmicutes*, *Bacteroidetes*, and *Actinobacteria* were the most prevalent microbial phyla, whereas *Streptococcus*, *Veilonella*, and *Prevotella* were the mainly enriched genera. As regards *Enterobacteriaceae*, a high relative abundance was observed at baseline, but it declined during antibiotic prophylaxis, reaching undetectable levels. However, patients with partial response showed a different trend, with a relative abundance of *Enterobacteriaceae* higher than 20% during quinolone administration, pointing to a possible therapeutic failure or lack of adherence to therapy [57]. In the previously mentioned study by Bajaj et al., SBP prophylaxis was able to increase Gram-positive bacteria belonging to *Lactobacillus*, *Enterococcus*, *Acidaminococcus*, *Akkermansia*, and *Pseudomonas* genera, and was also paralleled by an enrichment of CrAss phages as well as *Escherichia*, *Enterococcus*, *Shigella*, *Streptococcus*, and *Mycobacterium* phages, compared to patients with ascites who did not receive SBP prophylaxis. A drastic reduction in the complexity of the correlation networks with a positive linkage between *E. coli* and Gram-positive organisms was observed. Therefore, SBP prophylaxis increased the relative abundance of Gram-positive bacteria, due to the elimination of Gram-negative species [55]. Currently, there are no published studies demonstrating an association between long-term quinolone prophylaxis and bacterial resistances [35,61]. Alternative therapies for quinolone-resistant bacteria could be Trimetoprim–Sulfamethoxazole or Rifaximin [60]. In particular, rifaximin, a broad-spectrum antibiotic with negligible enteric absorption, has also been demonstrated to be effective in reducing the incidence of SBP [62,63]. The mechanism is linked to the reduction of intestinal endotoxemia, as well as bacterial attachment and internalization in enterocytes, and to its direct anti-inflammatory effect [64]. Rifaximin is considered an eubiotic rather than a simple antibiotic, as it does not alter the whole structure of the gut microbiota, promoting the growth of beneficial bacterial over pathogens, in a sort of “functional change”. In patients treated with Rifaximin, a reduction in *Veillonellaceae* and an increase in *Lactobacillus* spp. has been observed, as an effect of a beneficial modulation on the metabolic profile rather than a rearrangement of the composition of microbiota [65,66]. Moreover, Rifaximin seems to reduce *Streptococcus* abundance by targeting the phage–*Streptococcus* linkages, but further evidences are needed [67].

Bacterial species identified in the ascitic fluid of patients receiving Rifaximin are different from those identified in patients who did not receive antibiotic prophylaxis, as *Enterococci* and *E. coli* are usually more affected by the antibiotic therapy than other Gram-negative species (e.g., *Klebsiella* spp.) [62,63]. A recent study demonstrated that Rifaximin could be more effective than Norfloxacin in secondary prevention of SBP, as it acts also on quinolone-resistant bacteria [68]. Another randomized controlled trial comparing alternating treatment with Norfloxacin and Rifaximin versus Norfloxacin or Rifaximin alone as primary prophylaxis for SBP reported that the alternating schedule was more effective than Norfloxacin alone, but no significant difference was observed comparing Norfloxacin to Rifaximin alone [69]. Nevertheless, the use of Rifaximin for the prevention or treatment of SBP is still not recommended by guidelines, because the evidence from clinical trials is very weak [66].

## 5. Gut Microbiota Modulation as a Future Treatment for SBP

Since the gut microbiota plays a pivotal role in the onset and the progression of decompensated cirrhosis, its modulation might be used as a modifier of the natural course of the disease.

### 5.1. Probiotics, Prebiotics and Symbiotics

It has been reported that probiotic supplementation increases resistance to enteric infections in IL10-deficient mice and reduces endotoxemia in cirrhotic patients by modulating the viable counts of potentially pathogenic Gram-positive and Gram-negative bacteria in the intestine [70]. Based on this premise, clinical studies tested the administration of *Lactobacillus*-containing probiotics as a primary therapy in SBP, showing conflicting results. On one hand, *Lactobacillus* GG was not effective in preventing bacterial translocation and SBP, whilst the administration of *Lactobacillus johnsonii* plus antioxidants was able to obtain a reduction in bacterial translocation [7,71]; on the other hand, the addition of probiotics to norfloxacin did not improve the clinical efficacy in primary or secondary prophylaxis. Thus, the current literature does not support the use of probiotics to prevent the occurrence of SBP [72].

Prebiotics and symbiotics are commonly used to modulate imbalances in the gut microbiota [73]. It has been demonstrated that both prebiotics and symbiotics can reduce bacterial translocation from the gut [74]. Prebiotics have been found to increase the amount of occludin, claudin-3, and ZO-1, thereby restoring impaired tight junctions in the intestinal epithelium [75]. Given their impact on the gut microbiota and intestinal barrier, prebiotics and symbiotics could potentially be considered as an additional prevention strategy for SBP. However, to date, neither prebiotics nor symbiotics have been investigated for this purpose, or have been included in the guidelines for SBP management [76].

### 5.2. Enterosorbent Compounds and Bile Acids Signaling

An innovative strategy involves the absorption of intraluminal host or microbial metabolites or ligands, with the aim of reducing the translocation of pathogenic factors, such as ammonia or endotoxin, or modulating bile acid pathways [77]. Yaq-001 (Yaqrit Limited, Herefordshire, UK), a new non-absorbable synthetic carbon able to bind large molecular weight substances such as endotoxin, exotoxins, and cytokines, was found to improve liver injury, portal pressure, and LPS-induced ROS production in an in vivo model of cirrhosis [78]. Additionally, the phosphate sequestrant sevelamer is able to improve gut microbiota alpha-diversity and bind intraluminal endotoxin [79].

The administration of obethicolic acid, a farnesoid X receptor (FXR) agonist acting via bile acids signaling pathway, for 2 weeks to cirrhotic rats with ascites decreased bacterial translocation from 78.3% to 33.3%, significantly modulating mucosal microbiota composition and improving intestinal barrier function [7,79,80].

Although promising, more clinical studies are needed to evaluate the safety and tolerability of these compounds and their possible application in clinical practice.

### 5.3. Intestinal Motility

As the increased OCTT is considered one of the factors contributing to SIBO and bacterial translocation, the use of prokinetics such as cisapride, a 5-HT4-agonist, is supposed to reduce the incidence of SBP. An animal study analyzing the efficacy of cisapride and norfloxacin administration versus cisapride alone showed a reduction in the incidence of SBP in ascitic patients [81]. Another animal study demonstrated that rats treated with propranolol had a lower portal pressure, a shorter OCTT, and lesser occurrence of bacterial translocation and SIBO compared to rats receiving placebo, resulting in a decreased risk of SBP [82].

### 5.4. Other Experimental Treatments

Further ongoing studies are evaluating the effectiveness of bacteriophages, carbon nanoparticles-binding microbial components, and micro-RNA (as miR-320a against *Escherichia Coli*) treatments in reducing dysbiosis in patients with cirrhosis-related complications, but evidence on the efficacy of such treatments on SBP is expected in the near future [83].

## 6. Conclusions

SBP is a life-threatening complication of liver cirrhosis, which requires specific preventive measures and effective treatment. Although its pathogenesis is well known, with an established role of the gut–liver axis, therapeutic approaches aimed at improving gut dysbiosis and intestinal barrier permeability are not currently recommended by guidelines. An important point to consider, however, is the identification of predisposing factors; in fact, the assessment of intestinal permeability by urinary lactulose/mannitol ratio (MRL) or other methods [84] could be useful in identifying cirrhotic patients at risk of SBP who might benefit from close follow-up [43]. However, these tests are not universally available, are expensive and time-consuming, thus limiting their applicability in clinical practice. Another problem is that the treatment of SBP has remained stationary, being still based on broad-spectrum antibiotics, whereas it is clear that a fine modulation of the gut microbiota using more selective agents, such as bacteriophages, micro-RNA, and fecal microbiota transplantation, could be a winning one in terms of efficacy and unfavorable side effects for the patient, first and foremost the emergence of antibiotic resistance.

In conclusion, the alteration of the gut–liver axis is the hallmark of liver cirrhosis, which gives rise to many of the complications of the disease including SBP. Modulation of the gut microbiota, therefore, represents the focus of SBP treatment in the future, although scientific research in this field has not produced any new evidence recently, and further studies are needed to translate in practice the promising results of experimental models and preliminary clinical data.
